# The risk of revision after total hip arthroplasty in young patients depends on surgical approach, femoral head size and bearing type; an analysis of 19,682 operations in the Dutch arthroplasty register

**DOI:** 10.1186/s12891-019-2765-z

**Published:** 2019-08-22

**Authors:** M. F. L. Kuijpers, G. Hannink, S. B. W. Vehmeijer, L. N. van Steenbergen, B. W. Schreurs

**Affiliations:** 1 Radboud university medical center, Radboud Institute for Health Sciences, Department of Orthopaedics, Nijmegen, The Netherlands; 2 Radboud university medical center, Radboud Institute for Health Sciences, Department of Operating Rooms, Nijmegen, The Netherlands; 3 Department of Orthopaedic Surgery, Reinier de Graaf Hospital, Delft, the Netherlands; 4 Dutch Arthroplasty Register (Landelijke Registratie Orthopedische Implantaten), ’s-Hertogenbosch, the Netherlands

**Keywords:** Total hip arthoplasty, Young patients, Registry, Risk of revision

## Abstract

**Background:**

Total hip arthroplasty (THA) is used increasingly in younger patients. There is little knowledge about the effect of THA characteristics on risk of revision, especially in young patients. Therefore, we studied the influence of both patient-related and surgical factors on the risk of revision using data from the Dutch Arthroplasty Registry (LROI).

**Methods:**

All patients younger than 55 years with a primary THA implanted in the Netherlands between 2007 and 2017 were selected (*n* = 19,682). The covariates age, sex, primary diagnosis, ASA-classification, surgical approach, fixation method, bearing type, head size and year of surgery were entered into Cox proportional hazards models to calculate hazard ratios for the risk of revision.

**Results:**

The overall 5-year survival of primary THA was 95.3% (95% CI, 94.9–95.6). Use of the anterior approach resulted in a lower risk of revision than the use of the posterolateral approach (HR: 0.66, 95% CI: 0.47–0.92). THAs with a head diameter ≥ 38 mm had a higher risk of revision (HR: 1.90, 95% CI: 1.33–2.72) than THAs with 32 mm heads. Use of MoM bearings resulted in an increased risk when compared to C-PE (HR: 1.76, 95% CI: 1.27–2.43).

**Conclusion:**

The risk of revision in patients younger than 55 years depends on surgical approach, head size and bearing type. The anterior approach resulted in a decreased risk of revision, whereas use of ≥38 mm heads and MoM bearings resulted in an increased risk of revision for any reason.

## Background

Total hip arthroplasty (THA) has shown to be a cost-effective treatment for osteoarthritis of the hip, with reported increase in quality of life, regained physical ability and reduction of pain [1, 2]. THA is used increasingly in young patients, and this number will grow in the coming years. By the year 2030, it is estimated that more than 25% of all THA will be placed in patients under the age of 55 [3]. However, the outcome of THA in these young patients is inferior compared with older patients [4–6]. The main complications after THA include dislocation, infection, and loosening of the femoral or acetabular component. Young patients will outlive their prosthesis due to longer life expectancy, and survival at mid- and long-term is lower in patients younger than 55 years when compared with older patients [7].

Many studies reported factors that are associated with an increased risk of revision. For example, use of the posterolateral approach is associated with higher risks of revision due to dislocation and infection when compared with the anterior approach, and also resulted in more reoperations [8, 9]. Also the use of larger head diameters is associated with a lower risk of revision, where heads larger than 28 mm have a lower risk when compared to heads smaller than 28 mm [10]. However, these studies were not specific for young patients and the sample size of the described studies was low.

Understanding of factors that are associated with early revisions is of major importance, not only to be able to reduce the risks of revision, but also to provide realistic expectations to this young patient group.

Therefore, we studied the influence of both patient-related and surgical factors on the risk of revision due to any reason using data from the Dutch Arthroplasty Register (LROI).

## Methods

The LROI (Dutch Arthroplasty Registry) collects data about joint arthroplasties on a nationwide basis. Initiated by the Dutch Orthopaedic Association, data collection started in 2007. Coverage of all Dutch hospitals was reached in 2012. The database has a completeness of over 95% of primary THA and 88% for revision arthroplasty [11]. Patient characteristics are recorded at the moment of the primary procedure. Prostheses characteristics are derived from a implant library within the LROI, where all characteristics of prostheses used in the Netherlands are available [11].

For the present study, we included all patients younger than 55 years with a primary THA implanted in the Netherlands in the period between 2007 and 2017. Exclusion criteria were hip resurfacings, a surgical approach other than a posterolateral, anterior, direct lateral or anterolateral approach, and a bearing type other than ceramic-on-polyethylene (C-PE), metal-on-metal (MoM), ceramic-on-ceramic (CoC), metal-on-polyethylene (M-PE) and oxidised zirconium-on-polyethylene (Zr-PE).

### Statistics

Continuous variables were described using means and standard deviations, or medians and interquartile ranges, where appropriate. Categorical data were described using count and percentages.

Kaplan-Meier survival analyses were used to determine the 5-year survival rate with end-point revision for any reason. Cox proportional hazards models were used to analyze the influence of various covariates on the hazard ration (HR) of revision for any reason. The covariates age, sex, ASA-classification (I, II, III-IV), primary diagnosis, head size (22–26 mm, 28 mm, 32 mm, 36 mm and ≥ 38 mm), fixation method, surgical approach, bearing type (C-PE, MoM, M-PE, CoC and Zr-PE) and period of surgery (2007–2011, 2012–2016) were initially investigated as single covariates resulting in a crude HR with 95% CI with endpoint any reason. Primary diagnosis was dichotomized into primary osteoarthritis and secondary osteoarthritis. Secondary osteoarthritis results from a condition that changes the cartilage environment, including trauma, congenital or developmental joint abnormalities, metabolic defects and infection [12, 13]. Therefore, we included multiple diagnoses, such as dysplasia, osteonecrosis, (acute) fracture, inflammatory arthritis, late posttraumatic, post-Perthes and rheumatoid arthritis into secondary osteoarthritis. Multivariable Cox proportional hazard regression analyses were used to estimate adjusted HRs with 95% CI for endpoint revision for any reason, while adjusting for all mentioned covariates. Due to low numbers of events, we decided not to calculate the risk of revision for other endpoints than any reason to prevent violation of the events per variable ratio [14]. The proportional hazard assumption was checked for all variables added to the Cox proportional hazard model. All analyses were performed using R version 3.2.4 (R Foundation for Statistical Computing, Vienna, Austria).

## Results

### Patient- and implant characteristics

Between January 1st, 2007 and December 31st, 2016, a total number of 19,682 THAs were registered in patients under 55 years in the LROI. Mean age was 47.1 years (SD 7.33), more THAs were placed in women (53.3%). The most prevalent diagnosis was osteoarthritis (66.2%), and almost half of patients were ASA I (48.3%). Most THAs had an uncemented fixation (79.8%, *n* = 15,701), had a 32 mm head diameter (41.5%, *n* = 8165) and a C-PE bearing (48.2%, *n* = 9493). The posterolateral approach was used most frequently (62.8%, *n* = 12,367), followed by the direct lateral approach (20.4%, *n* = 4014), anterior approach (10.4%, *n* = 2053) and the anterolateral approach (6.3%, *n* = 1248). Patient- and implant characteristics are described in Table 1.

**Table 1:** Patient and implant characteristics of 19,682 THA in patients younger than 55 years old

	N (%)
Male	9127 (46.4)
Age (y)^a^	49.0 (45.0–52.0)
ASA-classification	
ASA I	9323 (47.4)
ASA II	8371 (42.5)
ASA III-IV	1368 (7.0)
Diagnosis	
Primary osteoarthritis	13,035 (66.2)
Secondary osteoarthritis	6453 (32.8)
Fixation	
Cemented	1924 (9.8)
Uncemented	15,701 (79.8)
Hybrid	312 (1.6)
Reversed hybrid	1614 (8.2)
Head Diameter	
22–26 mm	163 (0.8)
28 mm	5600 (28.5)
32 mm	8165 (41.5)
36 mm	4212 (21.4)
≥ 38 mm	750 (3.8)
Bearing type	
C-PE	9493 (48.2)
MoM	899 (4.6)
M-PE	3825 (19.4)
CoC	2701 (13.7)
Zr-PE	1132 (5.8)
Surgical approach	
Posterolateral	12,367 (62.8)
Anterior	2053 (10.4)
Direct lateral	4014 (20.4)
Anterolateral	1248 (6.3)

^a^Median (IQR)

### Reasons for revisions

The overall rate of revision was low. In total, there were 783 revisions 1 year after THA (3.98%). The most common reason for revision was dislocation (22.5%), followed by femoral loosening (18.0%), infection (16.9%) and acetabular loosening (14.9%) (Table 2).

**Table 2:** Reason for revision as percentage of all revisions

	N (%)
Dislocation	176 (22.5)
Loosening femur	141 (18.0)
Infection	132 (17.5)
Loosening acetabulum	117 (14.9)
Periprosthetic fracture	49 (6.3)
Other	208 (26.6)
Total	783 (100)

The total is more than 100%, as patients can have more than one reason for revision

The overall survival of primary THA with end-point revision for any reason at 1, 2 and 5 years follow up was 98.3% (95% CI: 98.2–98.5), 97.3% (95% CI: 97.1–97.6) and 95.3% (95% CI, 94.9–95.6), respectively.

### Unadjusted risk of revision for any reason

In the unadjusted Cox regression, a decreased risk of revision for any reason for the anterior approach was found. Additionally, also the direct lateral approach was associated with a decreased risk of revision. Furthermore, head diameters of 28 mm and ≥ 38 mm were associated with a higher risk of revision when compared to a head diameter of 32 mm. Finally, use of CoC as bearing type resulted in a decreased risk of revision, where use of MoM resulted in a significant increased risk of revision (Table 3).

**Table 3:** Risk of revision for any reason

	Crude HR (95% CI)	Adjusted HR (95% CI)^a^	*p*-value ^b^
Gender			
Female	1.02 (0.89–1.16)	1.09 (0.94–1.26)	0.27
Age	1.00 (0.99–1.01)	1.00 (0.99–1.01)	0.9
Year of surgery			
2007–2011	1	1	–
2012–2016	0.94 (0.82–1.09)	1.13 (0.96–1.35)	0.14
ASA-classification			
ASA I	1	1	–
ASA II	0.98 (0.85–1.13)	1.00 (0.85–1.16)	0.9
ASA III-IV	1.24 (0.97–1.60)	1.25 (0.94–1.64)	0.12
Diagnosis			
Primary osteoarthritis	1	1	–
Secondary osteoarthritis^c^	1.04 (0.91–1.20)	0.95 (0.81–1.12)	0.55
Surgical approach			
Posterolateral	1	1	–
Anterior	0.52 (0.38–0.71)	0.66 (0.47–0.92)	0.01
Direct lateral	0.82 (0.70–0.98)	0.86 (0.71–1.04)	0.12
Anterolateral	1.05 (0.82–1.34)	1.16 (0.89–1.52)	0.27
Fixation			
Uncemented	1	1	–
Cemented	1.15 (0.93–1.42)	0.91 (0.67–1.23)	0.54
Hybrid	1.24 (0.75–2.08)	1.32 (0.74–2.35)	0.35
Reversed hybrid	1.21 (0.96–1.52)	1.19 (0.91–1.55)	0.22
Head Diameter			
32 mm	1	1	–
22–26 mm	1.54 (0.79–2.99)	1.49 (0.75–2.99)	0.26
28 mm	1.21 (1.03–1.43)	1.11 (0.92–1.34)	0.28
36 mm	1.02 (0.84–1.23)	1.14 (0.92–1.42)	0.22
≥ 38 mm	2.85 (2.27–3.57)	1.90 (1.33–2.72)	< 0.001
Bearing type			
C-PE	1	1	–
MoM	2.39 (1.94–2.94)	1.76 (1.27–2.43)	< 0.001
M-PE	1.06 (0.88–1.27)	1.02 (0.83–1.26)	0.83
CoC	0.77 (0.61–0.97)	0.77 (0.60–1.00)	0.05
Zr-PE	0.92 (0.67–1.28)	0.90 (0.64–1.25)	0.52

^a^Multivariable Cox regression analysis performed with 17,288 observations and 741 events (complete cases); ^b^*p*-value based on adjusted HR; ^c^includes dysplasia, osteonecrosis, (acute) fracture, inflammatory arthritis, late posttraumatic, Post-Perthes and rheumatoid arthritis

### Multivariable risk of revision for any reason

**Fig. 1:**
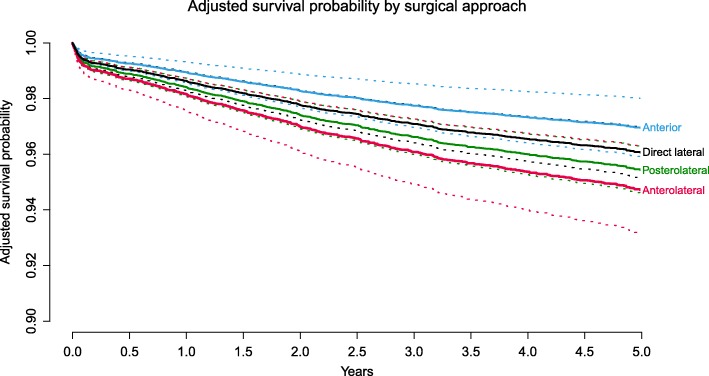
Adjusted survival probability by surgical approach

The use of the anterior approach resulted in a decreased risk for revision after THA when compared with the posterolateral approach (HR: 0.66, 95% CI: 0.47–0.92, *p* = 0.01). THAs placed using the direct lateral (HR: 0.86 (95% CI: 0.71–1.04, *p* = 0.1)) or anterolateral approach (HR: 1.16 (95% CI: 0.89–1.52, *p* = 0.3)) had no significant different risk of revision compared to the posterolateral approach (Table 3, Fig. 1).

**Fig. 2:**
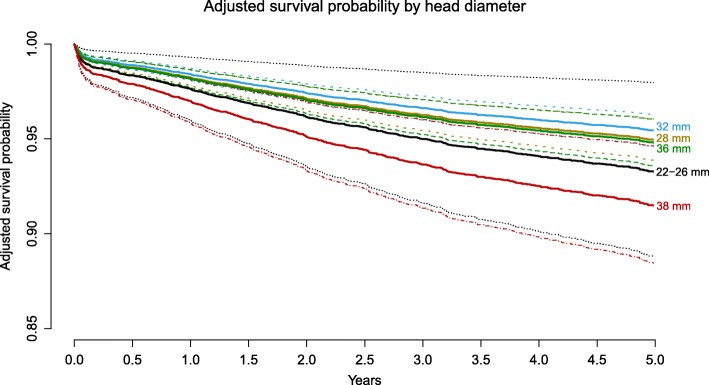
Adjusted survival probability by head diameter

The use of head diameters ≥38 mm resulted in an increased risk of revision when compared to 32 mm heads (HR: 1.90 (95% CI: 1.33–2.72, *p* < 0.001), Fig. 2). Additionally, the use of MoM bearings had an increased the risk of revision (HR: 1.76 (95% CI: 1.27–2.43, *p* < 0.001)).

## Discussion

The aim of this paper was to assess the influence of both patient-related and surgical factors on the short-term risk of revision in young patients, using data from the LROI. After adjusting for patient- and THA characteristics, our analysis shows a significant decreased risk of revision for any reason for the anterior approach. The use of the direct lateral approach and the anterolateral approach resulted in a reduced risk of revision, however these findings were not significant.

In literature, there is limited evidence about the effect of surgical approach on the short term risk of revision in young patients. Recent reports, assessing the short-term complication rate between the anterior and posterolateral approach, found no significant difference in postoperative complication rate and risk of revision [15, 16]. Some reports claim a favourable outcome of the anterior approach over the posterolateral approach when looking at recovery time and stability of the hip [17–19]. However, these studies did not focus on young patients and concluded that more evidence was needed.

We found an increased short-term risk of revision with increasing head diameters, which is in line with literature and registry reports [8, 20, 21]. In literature, the use of small head diameters is associated with an increased risk of dislocation [22, 23]. When we look at the rate of revision for dislocation in our population, we can conclude that the overall rate is low. Only 0.89% of all THAs were revised for dislocation 5 year after procedure, which is comparable with literature [24]. Due to these low numbers, we were not able to determine the effect of patient- and surgical characteristics on the risk of revision due to dislocation as endpoint.

It might be possible that the lower risk of revision of the anterior approach disappears after a longer follow-up. Another study based on LROI data evaluated the effect of surgical approach in all ages. [25]. In contrast to the current finding, they found the highest risk of revision for any reason for the anterior approach, even after exclusion of the first 150 procedures of each centre that performed the anterior approach to correct for a possible learning curve. Also other studies did not found an effect of surgical approach on a longer term [9, 26]. However, because of a higher activity level in young patients, and the high burden of young patients needing a revision procedure, it is of interest to analyze the short-term risk of complications.

Lastly, we found an increased risk for revision for MoM bearings. These findings are described extensively in literature and registry reports [20, 21, 27]. The use of other bearings did not result in significant differences when compared to C-PE.

In our study, we found low numbers of reported periprosthetic fractures, especially in the anterior group. A possible explanation might be a better bone quality and flexibility in young patients, which can result in less fractures after THA. However, a second explanation can be due to underreporting of periprosthetic fractures in the registry, where a reoperation with no replacements of any of the components of the implant, is not registered as a revision. A similar explanation would apply to infections, where treatment of infection without replacement of any of the components is not reported as a revision for infection in the registry [28]. Therefore, the actual percentage of revision, due to periprosthetic fractures and infections, might be higher than reported in this paper.

Because of the increasing use of the anterior approach, the effect of a learning curve for this approach should be addressed. De Steiger et al. concluded that 50 or more procedures need to be performed before the rate of revision is no different from performing 100 or more procedures [29], where the most reduction in complication rate occurred after the first 100 THAs [30]. Out of 100 institutes in the Netherlands, 27 performed at least 5 or more THAs using the anterior approach. Therefore, it can be concluded that the possible effect of a learning curve is present in our data. Despite this possible effect, the rate of revision was still lower for the anterior approach, when compared to all other approaches.

This study has some potential limitations that have to be considered. First, the follow-up of this study was limited. The effect of patient- and THA characteristics on risk of revision can change when follow-up is increased to a long term. However, especially for this young patient group, risk of revision on a short term is of major interest. Second, in our analysis we were unable to adjust for some variables that measure the patient’s demand on the implant, such as BMI and activity levels, as these were not, or only limited available from the Dutch Arthroplasty Register. Therefore, some residual confounding may be present. Lastly, we did not account for the possible effect of bilateral cases on the assumption of independence of observations in our statistical analysis. It has been shown that ignoring data dependency within a subject in studies involving bilateral cases may result in biased estimates [31, 32]. The extent of the resulting bias, however, was not determined in these studies.

Robertsson and Ranstam investigated if ignoring bilateral operations in statistical analyses biases the results, by analysing 55,298 prostheses in 44,590 patients using data from the Swedish Knee Arthroplasty Register [33]. They found that the effect of neglecting bilateral prostheses is minute, possibly because bilateral prosthesis failure is a rare event, and concluded that the revision risk of implants can be analysed without consideration for subject dependency, at least in study populations with a relatively low proportion of subjects having experienced bilateral revisions.

The percentage of bilateral implants in our cohort was 10.3%, which is considerably lower than the proportion of bilateral implants in the study of Robertsson and Ranstam (19.4%) [33]. Therefore, we think the possible effect of dependency between observations may be negligible in our analysis.

## Conclusions

In conclusion, there is a significant reduced risk of revision in patients younger than 55 years when the anterior approach was used. The use of head diameters ≥38 mm resulted in an increased risk of revision when compared with 32 mm heads, whereas the use of MoM as bearing type had also an increased risk of revision. The effect of THA characteristics is rarely evaluated in this young patient group. Understanding of risk factors is necessary to prevent early revisions, and manage expectations of young patients.

## Data Availability

Data are available from the LROI (Dutch Arthroplasty Registry) but restrictions apply to the availability of these data, which were used under license for the current study.

## Abbreviations

CoC: Ceramic-on-ceramic
C-PE: Ceramic-on-polyethylene
HR: Hazard ration
MoM: Metal-on-metal
M-PE: Metal-on-polyethylene
THA: Total hip arthroplasty
Zr-PE: Oxidised zirconium-on-polyethylene
